# Introduction to “Working Across Species”

**DOI:** 10.1007/s40656-018-0197-y

**Published:** 2018-04-24

**Authors:** Rachel Mason Dentinger, Abigail Woods

**Affiliations:** 1 Department of Undergraduate Studies, University of Utah, Sterling Sill Center, 195 Central Campus Dr, Salt Lake City, UT 84112 USA; 2 Department of History, King’s College London, Strand, London, WC2R 2LS UK

**Keywords:** Comparison, Animals, Medicine, Biology, Models, Analogy

## Abstract

Comparison between different animal species is omnipresent in the history of science and medicine but rarely subject to focussed historical analysis. The articles in the “Working Across Species” topical collection address this deficit by looking directly at the practical and epistemic work of cross-species comparison. Drawn from papers presented at a Wellcome-Trust-funded workshop in 2016, these papers investigate various ways that comparison has been made persuasive and successful, in multiple locations, by diverse disciplines, over the course of two centuries. They explore the many different animal features that have been considered to be (or else made) comparable, and the ways that animals have shaped science and medicine through the use of comparison. Authors demonstrate that comparison between species often transcended the range of practices typically employed with experimental animal models, where standardised practises and apparatus were applied to standardised bodies to produce generalizable, objective data; instead, comparison across species has often engaged diverse groups of non-standard species, made use of subjective inferences about phenomena that cannot be directly observed, and inspired analogies that linked physiological and behavioural characteristics with the apparent affective state of non-human animals. Moreover, such comparative practices have also provided unusually fruitful opportunities for collaborative connections between different research traditions and disciplines.

In January 2016, a geographically and disciplinarily diverse group of scholars gathered at King’s College London for a workshop entitled “Working Across Species: Comparative Practices in Modern Medical, Biological and Behavioural Sciences.” The workshop was convened under a Wellcome Trust-funded programme of research into the historical connections between human and animal health and medicine.^1^ Previous work performed under this programme by the authors and their colleagues had uncovered a raft of historically unacknowledged ways in which animals contributed to the development of modern medicine. This contribution rested to a significant extent upon the practice of interspecies comparison. We had identified scientists and doctors, working across a variety of spaces, disciplines and periods, who continuously compared and contrasted the anatomical structures, health conditions, physiological processes, and ecological impacts of diverse species in order to establish the nature and parameters of the relationships between them. These relationships were both biological (e.g., evolutionary relatedness) and health related (e.g., the likelihood of cross-infection by a particular pathogen or parasite). They were established within temperate and tropical countries, in medical laboratories, clinics, farms, forests, museums and zoological gardens, using animals that ranged from cows, sheep, and zebu, to rhinos, badgers, and monkeys (Woods and Bresalier 2014; Bresalier et al. 2015; Mason Dentinger 2016; Woods et al. 2018).

Struck by the dearth of scholarly engagement with interspecies comparison in the history of science and medicine, and intrigued by its varying methods, theoretical approaches, and the collaborations and networks it inspired, we decided to convene the “Working Across Species” workshop in order to explore further its forms, methods, and rationales. Ranging across fields from physiology to psychopathology and pharmacology, from the early nineteenth to the early twenty-first centuries, this topical collection samples the papers that were presented. Here, we introduce these papers and draw out some of their cross-cutting research questions: What qualified different species and organismic qualities as *comparable* and, conversely, how have human actors laboured, practically and theoretically, to *make* different animal species comparable? What methods and approaches have made for successful interspecies comparison? And, finally, how do practices of interspecies comparison enrich our understandings of the roles that animals have played in the history of science and medicine?

## Practising comparison

Each of the following papers gives a manifest sense of the scientific labour—both practical and theoretical—that has gone into enabling the drawing of inter-species comparisons. Often, the construction of elaborate experimental apparatus was necessary, whether these were large-scale mazes for sheep or tanks for the submersion of diverse species.^2^ Other times, the animal itself was refashioned via surgical intervention, as in the case of thyroidectomised sheep or pre-term piglets.^3^ The interventions used to make comparison possible frequently occurred in predictable sites of scientific research, such as medical laboratories and hospitals; but they also occurred in wholly unexpected locations, from the domestic setting (not to mention humanized clothing and habits) of a nineteenth-century orangutan in Paris to the stables of the horse-racing industry.^4^ Sometimes, scientists found it necessary to extract one animal from its typical setting and transplant it in a new context, which served to heighten contrasts and preconceived hierarchies between species even as researchers attempted to build comparative parallels between them. As Dam et al. (2018) recount, when a pre-term piglet was moved from a research laboratory to a hospital, its placement in an MRI machine intended for human patients instantly intensified awareness of its animal smell and appearance, both challenging and reinforcing notions of relatedness between pigs and humans in the process.

Yet, such forms of practical labour have been only one component of crafting comparison. Equally striking is the epistemic labour that goes into selecting qualities appropriate for comparison and justifying comparison between species that seem far removed from one another. Preconceived notions about the relatedness or evolutionary *proximity* between two species have suggested particular forms of comparison, based upon their shared evolutionary history (or lack thereof). As Burkhardt’s (2018) paper demonstrates, selecting and invoking such preconceptions could have profound implications for our understanding of humans: whether an orangutan’s brain and a human’s brain were considered different in their absolute quality or merely in size could indicate very different evolutionary and social lessons to be drawn from comparisons between the two. Ideas of ecological “proximity” suggested particular forms of comparison as well: Two species that were evolutionarily distant could still be compared based upon physiological or psychological adaptation to similar environmental features. For example, lack of oxygen is an ecological challenge common to a variety of species in very different environments. As Hagen’s (2018) paper demonstrates, using this challenge as a fulcrum for interspecies comparison suggested to physiologists a surprising range of eligible animal species, including fish, seals, sloths, and many more. A fish and a seal are both vertebrates, sharing a significant span of evolutionary history—yet, amongst vertebrates, they are only distantly related. Through considerations of ecological proximity, however, fish and seals were *made* comparable.

Sometimes, organismal features were made comparable by shifts in social values. In the case of twentieth-century pharmaceutical use in both horses and humans, as Worboys and Toon (2018) recount, biomedical comparisons between the two species became valid only after their relative social status shifted: In the mid-century, clinical observations of drug side-effects in horses and humans were not comparable, as horses were considered possessions rather than patients; but by the end of the century, the status of horses had changed, and increasing concern for their long-term health made potential health effects of drug use in humans and horses newly comparable. The shifting social value of nonhuman animals can also be seen in the paper by Dam et al. (2018). The biomedical researchers in their account employed a pre-term piglet as a surrogate for human neonates. Yet, the piglets’ equivalence to human infants evoked a sense of empathy in the researchers, creating a connection between researchers and subjects that at once generated emotional ambiguity and reinforced the very value of the experimental work being performed. In these two papers, the perceived degrees of humanness and non-humanness of horses and pigs shifted in line with human social values and emotional responses to them, which in turn validated or changed the types of comparisons that were considered to be scientifically appropriate.

In these ways, the papers show how one form of comparison often begat a different form of comparison. Through the experimental and theoretical labour of scientists, new qualities—both tangible and intangible—were made comparable. This was accomplished particularly well when a variety of methods and theoretical perspectives were engaged: In the case of Kirk and Ramsden’s paper, a single core researcher traversed the boundaries of physiology and psychology when drawing cross-species comparisons, while Dam, Sangild and Svendsen describe a diverse group of clinicians and biomedical scientists finding common ground in the physical, practical, and emotional connections they drew between preterm piglets and human infants. In the former, we can observe how the bases for comparison shifted over time, from physiological structures to psychological phenomena; in the latter, we see how multiple parties brought diverse perspectives and practices to bear on a single, wide-ranging study. In both cases (as reinforced by the collected papers as a whole), comparisons of anatomy, behavior, and disease were regularly deployed alongside each other, rather than being compartmentalized into specifically ecological, biological, and medical forms of investigation. Comparison thereby cut across disciplinary silos, its animal subjects acting as boundary objects that contributed to the simultaneous construction of diverse forms of scientific knowledge (Star and Griesemer 1989).

In addition, comparisons were often extrapolated outward by researchers, expanding the range of comparable features and feeding back upon and altering the preconceptions that warranted the comparative process in the first place. In other words, comparison was an iterative process: once a basis for comparison between two species was established, it often became a conduit by which increasing numbers of species or increasing numbers of organismic qualities could be justifiably compared. For Parisian anatomists in the nineteenth century, for example, comparisons of phrenological measurements of orangutan skulls and human skulls were only a starting point; “mental faculties” and artistic sensibilities became extensions of (and sometimes challenges to) these initial comparisons of more tangible organismal features.^5^

The comparison of both orangutan phrenology and mental faculties illustrates another feature common to many of the papers in this collection. Often, comparison of anatomical or physiological features, easily measurable and quantifiable, was used to validate the comparison of features we term here “intangible,” requiring the inference of more elusive qualities, such as intelligence and affect. In Kirk and Ramsden’s paper, for example, thyroidectomised sheep were objects of experimentation, originally employed to determine the role of thyroid function in various physical and physiological mechanisms; soon, however, they became individuals with psychologically significant affective states, comparable to those of humans. Psychobiologist Howard Liddell, who moved actively between the physiological and the psychological in his study of sheep, overcame his earlier doubts that inferences about animal psychological states lacked scientific rigor. In using comparison to consider relationships between different species, Liddell made explicit the link between the tangible and intangible, writing, “homology need not be limited to structures,” and could, in fact, be extended to behaviour.^6^ Similarly, Manias (2018) describes how palaeontologists’ comparisons between anatomical features of extinct and extant animal species were extended into comparisons of feeding behaviours, allowing the inference of ecologically adaptive behaviours in extinct animals, as well as their particular ecological environments, both long disappeared from the planet.

Examining the practices of interspecies comparison adds an important new dimension to our understandings of the roles that animals have played in the history of science and medicine. For decades, scholars have focussed their analyses on the use of animals in laboratory experimentation.^7^ While the intricacies of these practices continue to be analysed (Ankeny and Leonelli 2011), historians have typically understood the roles of animals in the biomedical laboratory in terms of their utility to humans. Animals could serve as surrogates for humans, for example in modelling the course of a disease or the result of a treatment regimen; as instruments for detecting or measuring biological processes, for example pregnancy diagnosis, which relied in the 1930s on mice responses to injections of women’s urine; and as culture media for generating biological products for use in humans, like the horses used to produce anthrax vaccines in the late nineteenth century.^8^ In all of these cases, efforts were made to standardise the animal participants, the products derived from their bodies, and the processes applied to them, with the goal of eliminating variation and achieving objective, generalizable outcomes. The papers in this topical collection, in contrast, explore a trajectory of animal research distinct from this narrowly biomedical experimental tradition. In casting a wider net, seeking manifold forms of comparison amongst a diversity of animal species, the scientists in these papers often decentred humans. Instead of attempting to press animals into the service of human health and medicine, they studied them as objects in their own right, and sought to comprehend and care for their variable bodies and psyches. In the case of Hagen’s (2018) paper, for example, *Homo sapiens* was but one in a diverse range of species employed to understand vertebrate physiological adaptation to extreme environments. Here and elsewhere, scientists did not seek resemblances between animals and humans that would allow the former to act as experimental proxies or surrogates of the latter. Instead, they practiced interspecies comparison, which often inspired attention to, or was predicated on the existence of differences between species.^9^ The resulting elucidation of the nature and implications of animals’ distinctive reactions to stimuli and environments shaped the development of scientists’ research programmes, their theoretical orientations, and how they cared for their animal subjects.

In fact, as a result of the emerging individual qualities of these animals, and their affective connections with human researchers, they were sometimes treated in experimental settings not as animal *models*, but as animal *patients*. This is a crucial distinction. The former implies an instrumentalist approach, in which regarding the animal as an individual patient would undermine the very validity of the research programme. By contrast, in a number of the papers in this collection, non-human animal subjects garnered forms of subjective observation and interaction that researchers strategically incorporated into the official protocols and broad conclusions of their research. For many of these researchers, empathy and care came to shape the trajectory of research. For the sheep in Kirk and Ramsden’s (2018) account and the pigs in Dam et al. (2018) account, affective connections between humans and nonhumans became incorporated into accepted scientific protocols.

## Papers in the “Working Across Species” topical collection

Beginning with Burkhardt’s (2018) account of the Muséum d’Histoire Naturelle’s acquisition of a live orangutan in 1836, the papers that follow lay the groundwork for an extended discussion of the ways that comparison has been used to structure scientific (and sociocultural) concepts of relationships between species. French anatomists of the era were sensitive to the potentially controversial transmutationist implications of comparisons between humans and other primates; yet they, like the Parisian public, were electrified by the likenesses they saw between their prize orangutan, Jack, and themselves. Burkhardt finds, in this volatile period between Lamarck and Darwin, that a strong sense of *proximity* came to the forefront in analyses of Jack’s more human traits, side-stepping the more exacting question of *ancestry* that would plague discussions of primate evolution after *On the Origin of Species* was published in 1859. *Proximity* provided the flexibility to consider differences of both kind and degree, alternately undermining and reinforcing the boundaries that divided humans from other primates. Set in the museum’s menagerie, the press and public made substantial contributions to this discourse, daring to draw comparisons between humans and orangutans that even Fréderic Cuvier (brother of Georges), with aspirations of establishing an explicitly *comparative* psychology, dared not broach—humanizing Jack and animalizing the humans around him with florid descriptions of their interactions. Burkhardt also sets the stage for a sustained discussion, continued throughout these essays, of the tension between the comparison of tangible and intangible organismal traits. For Cuvier and his contemporaries, for example, phrenological examination vied with estimations of the orangutan’s “force of reasoning” and “indefinable human character” as they attempted to ascertain the most reliable bases for interspecies comparison.

Manias’s (2018) account of an enigmatic and even “aberrant” family of extinct mammals, the Chalicotheres, plays upon a similar tension, considering how comparisons of anatomical features were supplanted or enhanced by comparisons of inferred ecological behaviours. Spanning a century, from initial fossil discoveries in the 1800s to the unearthing of more complete skeletons in the early twentieth century, Manias uses the Chalicothere to trace paleontological practices from earlier anatomical methods, such as Georges Cuvier’s “correlation of parts,” to later methods “reconstructing fossil animals as living, breathing animals within past ecological communities.” The Chalicothere’s “chimeric” anatomy seemed nonsensical and “incomparable” when considered through Cuvier’s lens; however, comparisons between the extinct organism’s hypothetical ecological behaviours and those of contemporary living animals, such as bears and okapi, made its otherwise disharmonious amalgamation of claws and hooves suddenly comprehensible. Here, then, Manias demonstrates the persistent value of comparison in palaeontology, even as the qualities compared and their implications have shifted over time. In the early twentieth century, the Chalicothere became newly *comparable* when its tangible anatomical features could be understood via analogy with the intangible, ecologically adaptive behaviours of extant organisms. Manias’ essay is the only one in the collection that does not consider comparisons between human and nonhuman animals and, as such, provides a valuable counterpoint to and confirmation of the other accounts: This history lacks the anthropocentric focus on “mental faculties” so dominant in the human–nonhuman comparisons; it shows, instead, yet another context in which comparisons of tangible organismal structures are used in conjunction and in competition with comparisons of intangible, context-specific behaviours.

This interplay between the tangible and intangible is also critical to Kirk and Ramsden’s (2018) essay on the comparative psychopathology of American Howard Liddell, working from the 1920s to the 1960s at the Cornell Behavior Farm. Liddell strove to understand pathological mental states in humans through the generation of analogous pathological states in other species of mammals. Trained in physiology and steeped in Pavlovian behaviourism, Liddell initially prized objectively measurable, quantifiable experimental data that could be tied directly to a demonstrable biological cause in the experimental animal’s body. He attempted to gauge the effects of surgical thyroidectomy on the “intelligence” of a wide range of animals, from the typical rats and dogs to the less typical pigs, sheep, and goats. However, years of this experimental work only exposed what Kirk and Ramsden call the “generative tension” at the heart of Liddell’s work: He came to recognize that a thyroidectomised animal’s behaviour could only be interpreted in terms of a context-dependent understanding of that animal’s normal range of behaviour within different environments. Liddell also began to consider the importance of intangible “emotional” factors in the lives of his subjects, viewing each animal as an individual, with its own subjective experience of the world and a recurring, unspoken appeal to him to “Remember—you are not studying physiology, you are studying me!” This recourse to subjective evaluation and explicit anthropomorphism was not, Kirk and Ramsden emphasise, an abandonment of Liddell’s scientific ideals to a weaker method of “speculation,” but was, instead, the forging of a new set of scientific ideals that embraced qualitative assessment and an “intimate” understanding of the nonhuman subject as *the* main conduit by which therapeutically useful comparisons could be made with human psychopathology. Though Liddell worked to create nonhuman animal models that could act as stand-ins for human patients, Kirk and Ramsden’s account upends the standard narratives of animal model creation, as Liddell’s practice follows the observations made by other authors in this collection, wherein comparative practices often exploit a diversity of species, eschew standardization, and emphasize individual experiences of whole animals responding to the diverse qualities of their varied environments—all aspects of Liddell’s work that run counter to our understandings of how experimental animal models of human physiology and behaviour were crafted in the early twentieth century.

Hagen’s (2018) essay explores contemporaneous research on the physiology of diving, finding an even more diverse group of experimental animals, from sloths to seals and mudskippers to manatees. Comparative physiologists Laurence Irving and Per Fredrik Scholander worked at various U.S. locations from the 1930s to the 1960s, devising experimental methods to gauge vertebrate species’ adaptations to oxygen deprivation. In the diversity of their subjects, Irving and Scholander saw evolutionary *unity*, using comparisons across species and across distinctive ecological contexts to argue for a vertebrate “master switch,” originating in deep time and branching outward to simultaneously explain a seal’s ability to survive underwater and a fish’s ability to survive above the water; in both cases, bradycardia (decreased heartbeat), reallocation of oxygenated blood to the brain, and anaerobic respiration in the muscles all contributed to the animals’ survival. The elegance of this comparison, which used diametrically opposed modes of existence to demonstrate physiological unity, illustrates again how many roles animals may play in experimental research; neither the seal nor the fish were a model, but both revealed fundamentally important physiological information about vertebrate animals, including humans. For Irving and Scholander, the implications for human physiology were important outcomes of the research (and, indeed, they collected data on blood oxygenation and breathing patterns in various humans, from pearl divers to professional swimmers). Yet, human concerns did not constitute the central objective of their research. The extrapolation of knowledge and methods from one species to the next relied on Irving and Scholander’s understanding of the unique ecological challenges that each species faced, rather than the creation of standardized conditions and organisms, which might have more easily produced knowledge directly translatable to humans.

Likewise, Worboys and Toon (2018) argue for the importance of context in their comparison of the use of the anti-inflammatory drug phenylbutazone, or “bute,” in horses and humans through the last half of the twentieth century. For humans, the long-term risks inherent in the use of bute created an unstable clinical picture from the drug’s introduction in the 1950s; by the 1980s, it had fallen into disrepute and was banned. The continued use in horses throughout this period, however, demonstrates the hierarchy of value placed on different animals’ lives and their quality of life. Humans constituted *patients*, who experienced the risky side-effects of taking bute. However, for decades, horses were mere human possessions and investments, and the impact and implications of bute use played out mainly in the realm of racing, where bute could create unfair competitive advantages; interpretation of clinical data suggesting potential health effects on horses were downplayed, thanks to the particular role that horses played in human life, while the differences between horse and human metabolism of the drug was emphasized. However, as the turn of the century neared, Worboys and Toon argue, this hierarchy subtly shifted and horses came, increasingly, to be seen as patients; thus, the clinical assessment of the human side-effects of bute began to influence perceptions of the use of bute use in horses, who were increasingly seen as patients themselves.

Dam et al. (2018) also observe fluidity in the hierarchy of values assigned to human and nonhuman animals in their ethnographic account of research on preterm neonatal piglets in a Danish translational medicine laboratory in 2013 and 2014. Like Kirk and Ramsden’s Liddell, their researchers aim explicitly to transform a barnyard animal into a model organism that can represent the human in a pathological state. And, intriguingly, like Kirk and Ramsden, they also find that subjective experiences and affective connections become crucial to the creation of successful comparisons between the humans targeted for therapy and the nonhuman animals used to model them. In fact, as Dam, Sangild and Svendsen observe, the attitudes of the “hybrid professionals” who move between the human clinical setting and the experimental pig setting suggest a tension between the necessity of standardized methods and data collection, and a style of individualized, devoted *human* care that would enable the piglets to more accurately model the human infants they represent in the laboratory. It is in this sense, then, that the hierarchy of value between humans and nonhumans is disrupted, as human clinicians argue for the individualized *care* of piglet *patients*. Yet, as Dam, Sangild, and Svendsen also recount, these shifts in value and affect, which appear at some moments to humanise the pigs and even lend them an aura of personhood in the eyes of the researchers, are also very effectively contained through the methods and standards enforced in the laboratory and the hospital beyond. After all, despite the emotive language of the researchers, only in the final sacrifice of the piglets can complete data be collected, an act that is inevitable in the laboratory context, and which makes the piglets’ difficult lives potentially valuable to future preterm human infants.

Taken together, these essays reveal the great potential for collaboration across scientific disciplines that comparison across species has offered (and continues to offer). But they also suggest that the work of creating comparison is highly reliant on the concerted efforts of researchers to either build upon or challenge preconceived notions of relationships between species, actively *making* species and their characteristics *comparable*. Such practices usually began with ideas of evolutionary proximity and anatomical or physiological resemblance between species. But these initial premises were often transformed as awareness of animals’ particular ecological environments and responses to behavioural stimuli grew, or as a result of shifting cultural assumptions, social values, and personal experiences with animals. In particular, the degree to which cross-species comparison created subjective and inferential comparisons between humans and nonhumans is striking. Eschewing standardization and rejecting the valorisation of supposed objectivity, “working across species” instead often highlighted the usefulness of subjective connections between species. The capacity to create such connections, the exploitation of diversity, the ability to make theoretical and practical use of both similarity and difference, and the movement of researchers and modes of inquiry across disciplinary boundaries—all of these appear to mark the most effective and compelling examples comparison, as well as opening many further avenues for scholarly inquiry.

## Footnotes

1 “One Medicine? Investigating human and animal disease”; Programme Grant reference: 092719/Z/10/A; Principal investigator: Abigail Woods; Postdoctoral fellows: Michael Bresalier, Angela Cassidy, Rachel Mason Dentinger, and Kathryn Schoefert.

2 For mazes: Kirk and Ramsden (2018); For tanks: Hagen (2018).

3 For sheep: Kirk and Ramsden (2018); for pigs: Dam et al. (2018). Also see Woods (2018).

4 For a nineteenth-century orangutan: Burkhardt (2018); horse racing: Worboys and Toon (2018).

5 In Burkhardt (2018) the projection of musical taste on the Parisian orangutan became part of the comparison between the orangutan and human.

6 Kirk and Ramsden (2018: 20), quoting Liddell (manuscript, untitled response to criticism from Dr. Bloomberg and Dr. Foley, p.1, n.d. c.1950s. HSL, Box 5, Folder 43.).

7 See the following selected texts from an otherwise very large literature: Bynum (1990), Clause (1993), Löwy and Gaudillière (1998), Löwy (2003), Slater (2005), Rader (2007), Kirk (2008) and Franco (2013).

8 On the distinction between animal models as biomedical replica and animal models as instruments, see Germain (2014). For the use of mice in pregnancy testing, see Olszynko-Gryn (2013). For the use of animal bodies to produce vaccines and sera, see Latour (1988), Simon (2008), Kotar and Gessler (2013), Mendelsohn (2016).

9 With the particular exception of Dam et al. (2018), in which the research is pursued explicitly for the benefit of human infants and relies upon resemblance between the human infant and piglet model.
